# Effect of deletion of gene cluster involved in synthesis of Enterobacterial common antigen on virulence and immunogenicity of live attenuated *Salmonella* vaccine when delivering heterologous *Streptococcus pneumoniae* antigen PspA

**DOI:** 10.1186/s12866-020-01837-0

**Published:** 2020-06-08

**Authors:** Qing Liu, Xuegang Shen, Xiaoping Bian, Qingke Kong

**Affiliations:** 1 College of Animal Science and Technology, Southwest University, Chongqing, China; 2 Chongqing Engineering Research Center for Herbivores Resource Protection and Utilization, Chongqing, China

**Keywords:** ECA, *Salmonella* Typhimurium, Virulence, Vaccine vector, Th2 immunity

## Abstract

**Background:**

Enterobacterial common antigen (ECA) is a family-specific surface antigen shared by all members of the Enterobacteriaceae family. Previous studies showed that the loss of ECA results in *Salmonella* attenuation, indicating its usefulness as a vaccine candidate for *Salmonella* infection, but no studies have shown whether the mutation resulting from the deletion of the ECA operon in conjunction with other mutations could be used as an antigen vehicle for heterologous protein antigen delivery.

**Results:**

In this study, we introduced a nonpolar, defined ECA operon deletion into wild-type *S.* Typhimurium χ3761 and an attenuated vaccine strain χ9241, obtaining two isogenic ECA operon mutants, namely, χ12357 and χ12358, respectively. A number of in vitro and in vivo properties of the mutants were analyzed. We found that the loss of ECA did not affect the growth, lipopolysaccharide (LPS) production and motility of *S.* Typhimurium wild type strain χ3761 and its attenuated vaccine strain χ9241 but significantly affected the virulence when administered orally to BALB/c mice. Furthermore, the effects of the ECA mutation on the immunogenicity of a recombinant *S.* Typhimurium vaccine strain χ9241 when delivering the pneumococcal antigen PspA were determined. The result showed that the total anti-PspA IgG level of χ12358 (pYA4088) was slightly lower than that of χ9241 (pYA4088), but the protection rate was not compromised.

**Conclusions:**

ECA affects virulence and benefits the Th2 immunity of *Salmonella* Typhimurium, therefore, it is feasible to use a reversible ECA mutant mode to design future *Salmonella* vaccine strains for heterologous protective antigens.

## Background

Enterobacterial common antigen (ECA) is a kind of unique glycolipid on the cell surface of all *Enterobacteriaceae* family members, such as *Klebsiella*, *Proteus*, *Shigell*a, *Yersinia*, and *Salmonella* [1–4]. It consists of linear repetitive units of a trisaccharide composed of 4-acetamide-4,6-dideoxy-D-galactose (Fuc4NAc), N-acetyl-D-mannosaminuronic acid (ManNAcA), and N-acetyl-D-glucosamine (GlcNAc). It is considered the second dominant immunogen, ranked next to the lipopolysaccharide (LPS) O-antigen [3, 5, 6]. Three ECA variants, ECA_PG_, ECA_LPS_ and ECA_CYC_, have been described since it was first found in *E. coli* [3]. ECA_PG_ is the major form that is linked to diacylglycerol and the only type of ECA present in *Salmonella* [7]; ECA_LPS_ is anchored to the lipid A core region of LPS [8]; and ECA_CYC_, which contains four or six trisaccharide repeat units, is a water-soluble cyclic form, not expressed on the surface of bacteria but in the periplasmic space [2, 9]. ECA plays important roles in virulence in the *Enterobacteriaceae* family because of its impacts on bile resistance, motility and other characteristics [10–13]. Similar with O-antigen, ECAs are also polysaccharide antigens anchored on the surface of bacteria cells and can trigger the production of anti-ECA antibodies in mice. Nevertheless, these anti-ECA antibodies hardly account for the passive immune protection against these *Enterobacteriaceae* strains, which distinguish from other virulence factors and surface polysaccharides. Furthermore, these anti-ECA antibodies can escape from the host immune system and last over a long period of time in the bodies of hosts, such as patients with shigellosis, peritonitis [14], chronic urinary tract infections (UTIs) [15] or chronic pyelonephritis [14–16]. The avoidance of triggering anti-ECA antibody production in the host by the *Salmonella* vaccine may contribute to increased stimulation of the immune response against heterologous antigens.

**Fig. 1:**
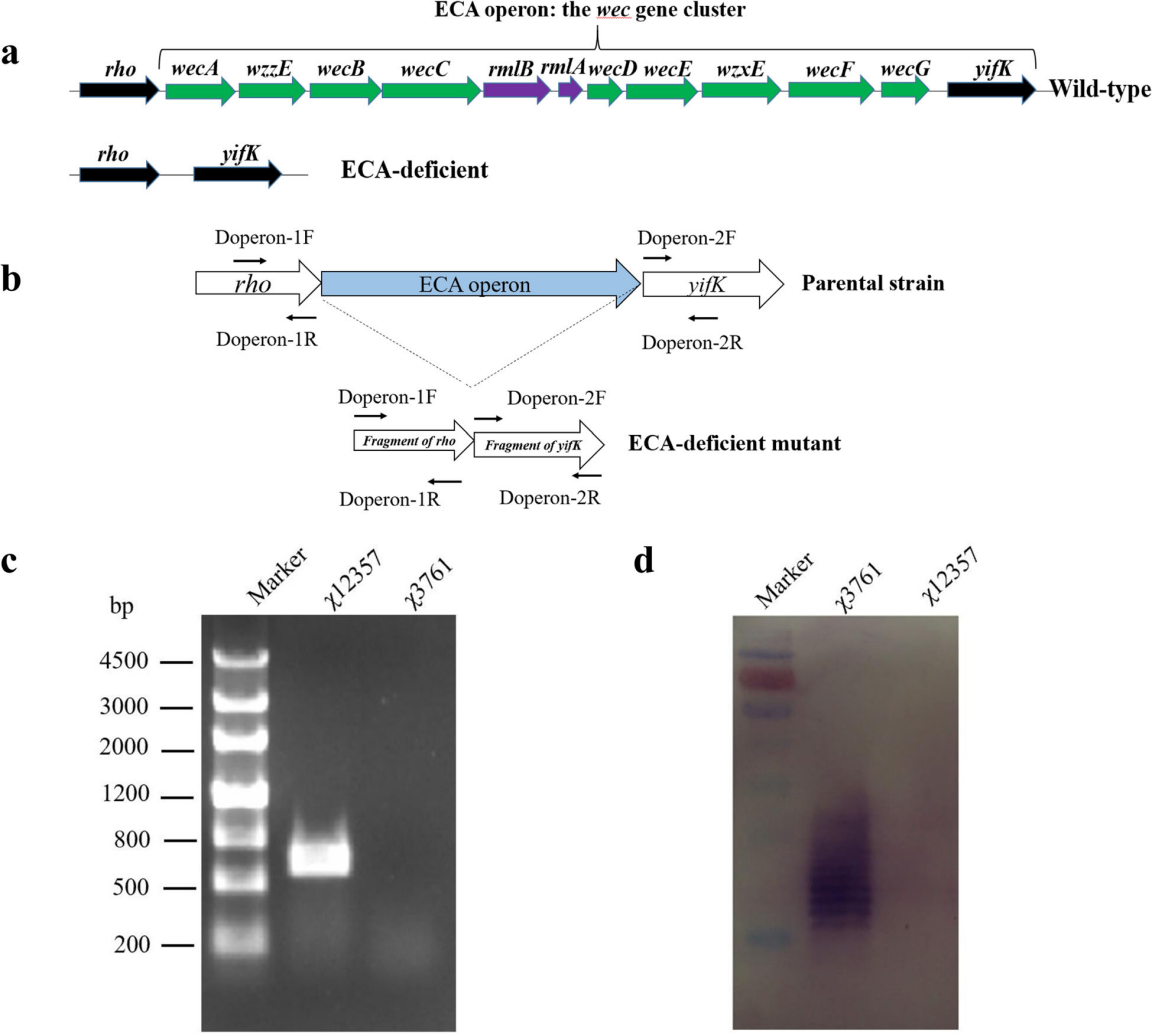
ECA operon deletion in *S*. Typhimurium χ3761 **a** Map of the ECA operon. **b** Schematic strategy for deletion of the ECA operon by allelic exchange using suicide vectors; the whole ECA operon from *wecA* to *wecG* was deleted. **c** ECA operon deficiency mutation identification by PCR amplification. Primer pairs for Doperon-1F/Doperon-2R were used to amplify the ECA operon using the ECA-deficient *S*. Typhimurium mutant χ12357 (lane 1) and the wide type χ3761 (lane 2) as the PCR template. A PCR product of approximately 800 bp was observed for χ12357, but no bands were observed for wild type *S*. Typhimurium χ3761. **d** Detection of ECA using ECA-specific mAb898 antibody by western blotting. The wide type *S*. Typhimurium χ3761 could produce ECA, but the ECA-deficient *Salmonella* strain χ12357 lost the ability to produce ECA

The genetic determinants of ECA_PG_ are located in the *wec* (formerly *rfe*) gene cluster from *wecA* to *wecG* (Fig. 1a), which is responsible for the synthesis of ECA polysaccharides, the addition of ECA polysaccharide chains to the lipid carrier and the transportation of ECA to the bacterial cell surface [11, 17, 18]. The inactivation of specific genes in this gene cluster results in the loss of ECA polysaccharides in *Enterobacteriaceae* family members and attenuates these pathogens. For example, the *wecA* mutation in *Haemophilus ducreyi* 35000HP impairs pustule formation in humans, and the *wecE* mutation in uropathogenic *E. coli* (UPEC), which results in dysfunction in the synthesis of ECAs, attenuates the effects of murine urinary tract infection [19, 20]. In *Salmonella* Typhimurium, Δ*wecA* mutants are also more sensitive to bile and less lethal to mice. Due to the undesirable host immune response stimulated by ECAs, recent studies have revised the ECA of the *Salmonella* vaccine carrier to deliver O-antigen polysaccharide [21, 22]. We demonstrated that downregulation of O-antigen expression could reduce the immune response against specific O-antigen of attenuated *Salmonella* carriers while triggering a strong immune response against heterologous antigens [23–25], and downregulation of both O-antigen and ECA production in *Salmonella* enhances the immunogenicity and cross-protective efficacy against heterologous *Salmonella* challenge [26]. Therefore, we speculate that the removal of ECA from live attenuated *Salmonella* Typhimurium vaccine strains would expose more heterologous antigens and outer membrane proteins to the host, leading to a greater immune response to the heterologous antigens and better protective efficacy.

The ideal live *Salmonella* vaccine carriers show attenuated virulence but an enhanced immune response against the heterologous antigens delivered by vaccine carriers. In our previous studies, a regulated delayed expressed system (RDES) in a Recombinant live recombinant attenuated *Salmonella* vaccine (RASV) strain was designed to deliver heterologous antigens regulated by arabinose. In this RDES, there are two components: RASV χ9241-carrying mutated genes of Δ*pabA* Δ*pabB* Δ*asdA* Δ*relA198*::*araC* P_BAD_*lacI* TT deletion/insertion (TT stands for transcriptional terminator) and a plasmid pYA4088 that consist of a recombinant *Streptococcus pneumoniae* antigen *pspA* gene fused to DNA encoding the β-lactamase signal sequence for directing recombinant PspA to the periplasmic region [27]. As an attenuated live vaccine strain, χ9241(pYA4088) invades and colonizes host tissues by oral immunization. The presence of arabinose positively regulates the synthesis of LacI, which can prevent PspA expression by binding to the P_trc_ promoter on pYA4088. In contrast, no LacI is expressed without arabinose in the growth environment of strain χ9241(pYA4088), resulting in production of the heterologous PspA that are carried on pYA4088. This regulated delayed expressed system has been widely used to evaluate the effects of other mutations on immunogenicity against heterologous antigens [28–30]. In this work, we constructed an ECA-deficient *Salmonella* Typhimurium mutant by deleting the entire ECA operon from *wecA* to *wecG* based on the wild type strain χ3761 [31] and the attenuated live vaccine strain χ924 1[32], and evaluated the effect of removing the whole ECA operon on the virulence and immunogenicity of wild type *Salmonella* Typhimurium and its attenuated mutants. We also tested the protective efficacy of the generated mutants in BALB/c mice infected with *Streptococcus pneumoniae.*

## Results

### Construction of the ECA-deficient *Salmonella* mutant strain

In the *S.* Typhimurium wild type strain UK-1 χ3761 and its attenuated vaccine strain χ9241, the ECA operon consisted of a series of *wec* genes, ranging from *wecA* to *wecG*, as stated in Fig. 1a. We deleted the whole ECA operon of the wild type strain UK-1 χ3761 and the attenuated vaccine strain χ9241 and generated two ECA defective mutants, χ12357 and χ12358, by suicide plasmid-mediated homologous recombination (Fig. 1b). A pair of primers, Doperon-1F/Doperon-2R, were used to amplify the ECA operon from χ 3761 and χ12357, and a PCR product of approximately 800 bp could be observed for χ12357, but no bands were observed for χ3761 in agarose gel, which indicated that mutants with deletion of the ECA operon (from *wecA* to *wecG*) were created in χ12357 (Fig. 1c). Furthermore, immunoblotting using the mouse anti-ECA monoclonal antibody mAb898 demonstrated deletion of the EAC operon, showing the ECA-negative phenotype [33] (Fig. 1d). Following the same method, we also deleted the ECA operon in the attenuated vaccine strain χ9241 and created an ECA defective vaccine strain χ12358 (data not shown).

### Phenotypic characterization of the ECA deficient mutants

**Fig. 2:**
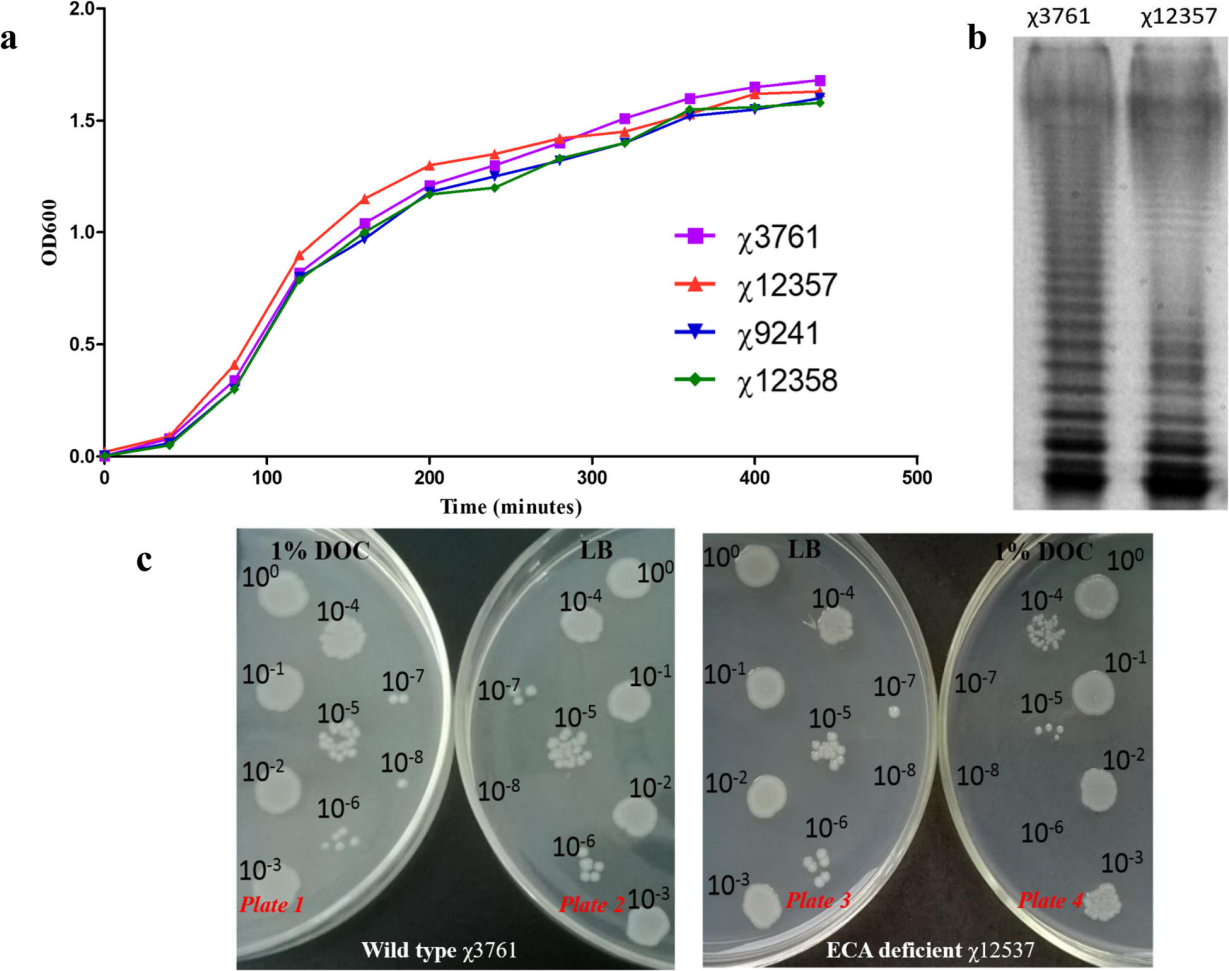
Phenotypes of the wide type *S*. Typhimurium and ECA-defective mutant strain. **a** Growth curves. Overnight cultures were diluted to an optical density of 0.025 and incubated at 37 °C with shaking at 180 rpm; the OD_600_ was measured at the appropriate time. **b** Bile sensitivity assay. Three-microliters of a 10-fold dilution of the overnight cultures of the wide type χ3761 and mutant χ12357 strains were deposited and incubated for 12 h at 37 °C in LB plates with or without 1% DOC. **c**. LPS profiles. LPS from χ3761 and the mutant χ12357 were subjected to 12% SDS-PAGE and then silver stained

The growth rate of the mutants was evaluated in LB broth and showed that the growth curves of the ECA-deficient mutants χ12357 and χ12358 were similar to those of their parental strains χ3761 and χ9241. Both χ3761 and χ12357 reached the end of the log phase with an OD_600_ of 1.5 for approximately 5 h, while the vaccine strain χ9241 and its derivative strain χ12358 had slightly lower rates of growth due to the deletion of a few virulent genes, such as *pabA*, *pabB*, *araBAD* and *relA* (Fig. 2a). The LPS profile of the ECA-deficient mutant χ12357 was indistinguishable from that of the parental wild type strain χ3761, indicating that ECA deficiency has no effect on the synthesis of LPS in *S*. Typhimurium (Fig. 2b). To determine the effect of ECA deficiency on bile resistance, drops of serial dilutions of χ3761 and χ12357 strain suspensions were incubated on LB plates with or without 1% deoxycholate (the main active ingredient of bile, DOC). The growth state of χ3761 and χ12357 appeared similar on regular LB plates (plates 2 and 3). A few colonies were observed on the 10^7^ dilution drops of both strain suspensions, suggesting no differences in the growth rate and susceptibility between the two strains on LB plates. Nevertheless, their growth states were totally different on the LB plates containing 1% DOC (plate 1). For the wild type strain χ3761, a few colonies grew on the 10^7^ dilution, and the same growth tendency as that of the regular LB plate was observed, suggested that 1% DOC had no effect on the growth of the wild type strain; however, the 10^5^ dilution of the χ12357 suspension grew a few colonies, suggesting that the *wec* operon mutants were significantly suppressed by the presence of 1% DOC on plate 4 (Fig. 2c). Moreover, under the high concentration DOC bile condition, 87.6% of the *wec* operon χ12357 mutants were also killed, but more than 90% of the wild type strains remained viable (Table 3). To assess the relationship between ECA and motility, the ECA-deficient mutant χ12357 and wild type χ3761 strain were dropped onto 0.3% agar plates. Both the ECA-deficient mutants and wild type strains were able to swim on soft agar, with no defects in motility (Table 1).

**Table 1:** Motility, DOC sensitivity and virulence of the wild-type *S.* Typhimurium χ3761 and its ECA-deficient mutant

Strain	Motility (cm) on soft agar	Death rate (%) (DOC)	LD_50_
χ3761	6.2 ± 0.1	9.5	2 × 10^4^
χ12357	6.4 ± 0.1	87.6	> 10^9^

### Virulence and colonization of the mutant strain in mice

**Fig. 3:**
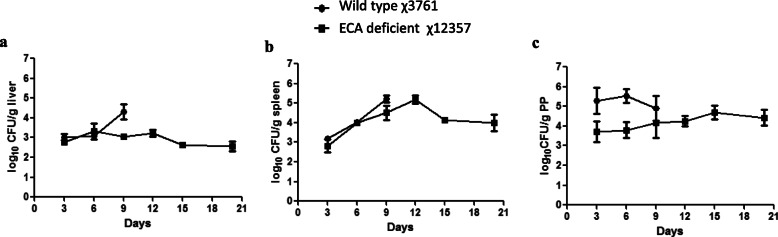
Bacterial colonization in mouse organs. Groups of mice (3 mice/group) were orally inoculated with approximately 1 × 10^9^ CFU of the indicated strains. The bacterial loadings (log CFU/g) in the tissue were determined on days 3, 6, 9, and 12 post-inoculation in the liver (**a**), spleen (**b**), and PP (**c**). The horizontal lines represent the means, and the error bars represent the standard errors of the means. All the mice in wild-type χ3761 group died at 9 days after oral inoculation

The virulence of the ECA-deficient mutant was assessed by determining the oral LD_50_ of the strains in BALB/c mice. The results showed that χ3761 was highly virulent with an LD_50_ of 5 × 10^4^ CFU, whereas the ECA-deficient mutant χ12357 was highly attenuated and no deaths were observed following infection of the mice at the highest tested dose (1.0 × 10^9^ CFU), which suggested that the *wec* operon mutation made the wild type *S*. Typhimurium avirulent (Table 1). Further, the capability of the ECA-deficient mutant χ12357 to efficiently disseminate and colonize mouse tissues was assessed by determining the bacterial loading in the organs of the mice, including the liver, spleen and Peyer’s patch, post-infection (Fig. 3a, b, c). Consistent with the previous result, all of the mice inoculated with χ3761 succumbed to infection within 9 days post-infection, and all of the mice inoculated with χ12357 survived until 21 days post-infection and most of them appeared health and active. The colonization data showed that the bacterial counts in the organs from the mice inoculated with the wild-type strain rapidly increased, and bacterial loading in the organs of the wild-type-inoculated mice was significantly higher than that of the mice inoculated with the ECA-deficient mutant within 9 days post-infection. Further, the ECA-deficient mutant also colonized well in the mice organs and maintained a moderate level of bacterial loading, with persistent infection throughout the experiment. These data demonstrate that the ECA-deficient mutant was able to efficiently colonize the mice and establish a persistent infection, consistent with the results of a previous study [18].

### Expression of the pneumococcal antigen PspA in the *Salmonella* strains

Attenuated *Salmonella* χ9241 carrying Δ*pabA* Δ*pabB* mutations is often used in our lab to evaluate the effects of other mutations on immunogenicity [24]. To assess the effects of ECA deletion on immunogenicity in attenuated *Salmonella*, we introduced the *wec* operon mutation into χ9241 to yield strain χ12358. Moreover, the *asd* recombinant plasmid pYA4088, which carries a recombinant *pspA* gene fused to the DNA encoding the β-lactamase signal sequence to direct PspA to the periplasmic region, was introduced into both χ9241 and χ12358. Both strains (χ9241 and χ12358) were grown in LB medium with or without 0.1% arabinose, and western blot analyses were used to determinate the synthesis levels of PspA in the above bacterial cells. No PspA was detected in χ9241 (pYA3493, carrying the empty vector, which served as a negative control) regardless of whether it was grown in the presence of arabinose. Compared with the negative control strain χ9241 (pYA3493), both strains (χ9241 and χ12358) carrying pYA4088 produced PspA when arabinose was absent, and instead expressed LacI in the presence of arabinose (Supplementary Fig. 1). This result suggests two points: the regulated delayed expressed system was successfully used to construct the attenuated vaccine vector strains χ9241 (pYA4088) and χ12358 (pYA4088); and the *wec* operon mutant χ12358 has a similar ability to express heterologous antibodies, such as PspA, as its parent strain χ9241.

### Immunogenicity of the *wec* operon mutants after oral administration

**Fig. 4:**
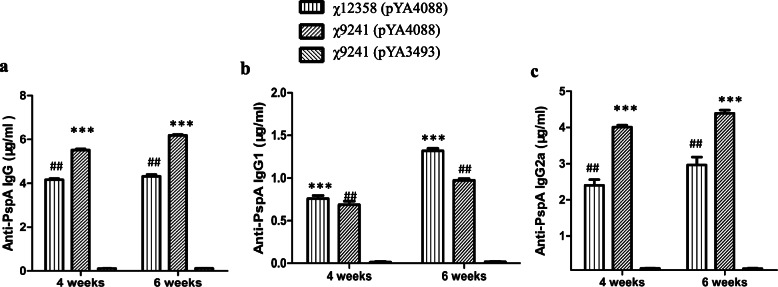
Serum responses and cross-reactivity. Total serum IgG specific for PspA (**a**), IgG1 specific for PspA (**b**) and IgG2a specific for PspA (**c**) in mice immunized with χ9241 (pYA4088) or χ12358 (pYA4088) were measured according to standard antibodies by ELISA. The error bars represented variations between triplicate wells. Mice were boosted at week 4. ***, *P* < 0.01 compared to the concentration from the mice immunized with χ9241 (pYA3493). ##, P < 0.01; ###, *P* < 0.001 compared to the concentration from the mice immunized with χ9241 (pYA4088)

To assess the immunogenicity of the *wec* operon mutants expressing heterologous antigens as vaccine vectors, three groups of mice were immunized and boosted with 1 × 10^9^ of χ12358 (pYA4088), χ9241 (pYA4088) or χ9241 (pYA3493). Serum IgG, IgG1 and IgG2a antibody responses to PspA in sera collected at week 4 and week 6 were measured by ELISA. Compared with the negative control strain χ9241 (pYA3493), both χ12358 (pYA4088) and χ9241 (pYA4088) significantly stimulated mice to produce high levels of IgG, IgG1 and IgG2a anti-PspA antibodies, suggesting that the deletion of the *wec* operon had no effect on the immunogenicity of the vaccine strains. Noticeably, χ12358 (pYA4088) induced levels of anti-PspA IgG1 isotype antibodies that were significantly higher than that of χ9241 (pYA4088) (*P* < 0.001), but the levels of total IgG and IgG2a isotype antibodies in the mice induced by χ12358 (pYA4088) were significantly lower than that induced by χ9241 (pYA4088) (Fig. 4a, b, c).

### Evaluation of immunogenicity against conserved OMPs from different enteric bacteria

**Fig. 5:**
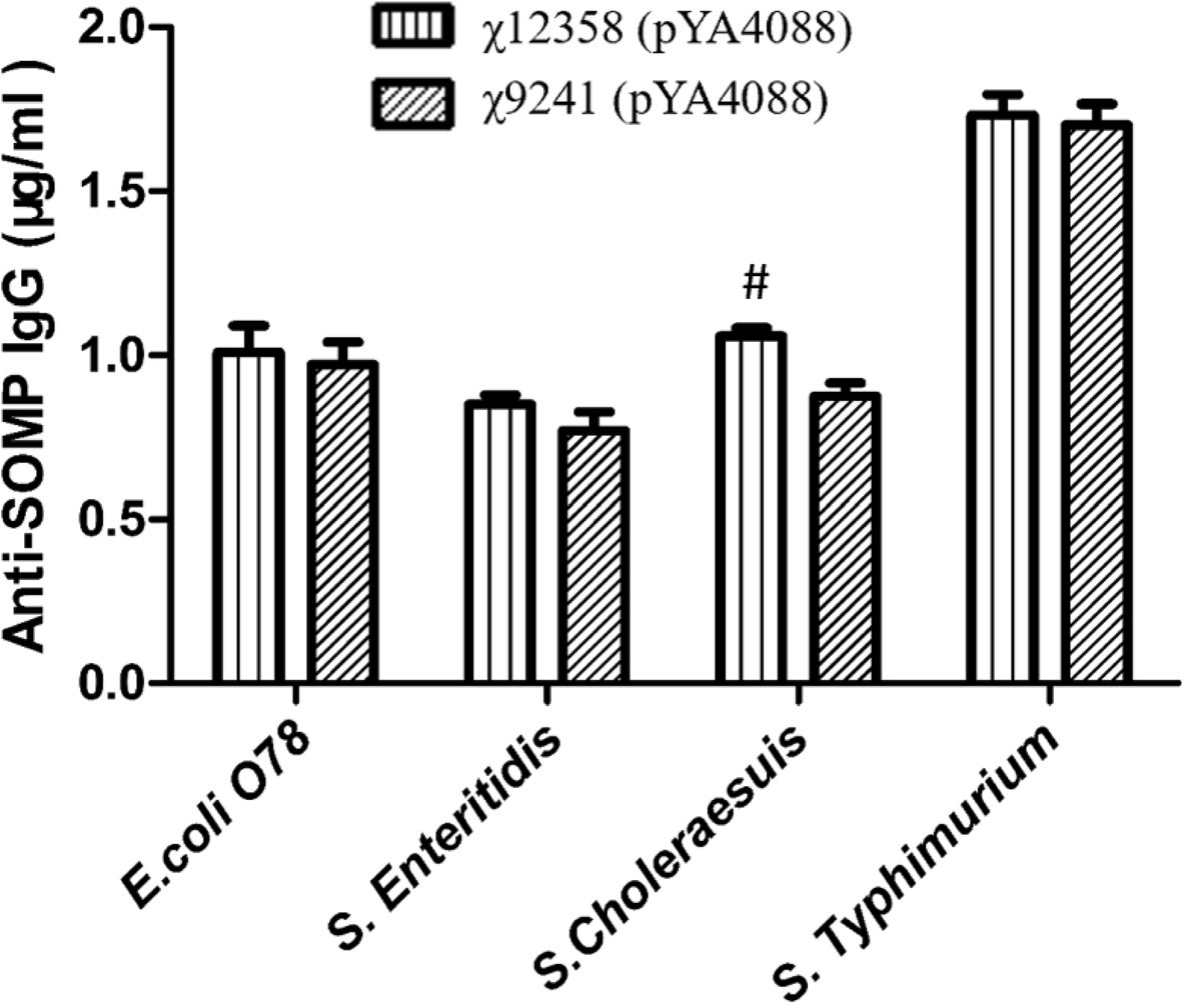
Serum responses and cross-reactivity. IgG levels of sera obtained from mice orally immunized with χ9241 (pYA4088) or χ12358 (pYA4088) to homologous and heterologous SOMPs were measured. The error bars represented variations between triplicate wells. Mice were boosted at week 4. ***, P < 0.01 compared to the concentration from the mice immunized with χ9241 (pYA3493). ##, P < 0.01; ###, P < 0.001 compared to the concentration from the mice immunized with χ9241 (pYA4088)

To evaluate the cross-reactivity of the serum antibodies raised from mice orally immunized with the mutant χ12358 (pYA4088) against outer membrane proteins (OMPs) purified from several different homologous and heterologous wild-type enteric bacterial strains, including *S*. Typhimurium (group B), *S*. Choleraesuis (group C1), *E. coli* O78 and *S*. Enteritidis (group D1), we measured the IgG antibody responses to OMPs from the above pathogens in serum collected at week 6 after the mice were immunized with χ12358 (pYA4088) and χ9241(pYA4088). The IgG level of cross-reaction against OMPs from *S*. Typhimurium (group B), *E. coli* O78 and *S*. Enteritidis (group D1) stimulated by χ9241 (pYA4088) and χ12358 (pYA4088) were not different, but the level of IgG against OMPs from *S*. Choleraesuis stimulated by the *wec* mutant χ12358 were significantly higher than that induced by χ9241 (pYA4088) (Fig. 5). This result was consistent with a previous study, in which a lack of or the regulated synthesis of dominant surface antigens, such as the O-antigen of LPS, increased the serum antibody cross-reactive to conserved surface OMPs of other enteric bacteria [24, 36].

### Comparison of the protective efficacy of live attenuated vaccine strains and their *wec* operon mutants

To test whether the *wec* operon mutation affects the protective efficacy of RASVs against pneumococcal infection, BALB/c mice orally immunized and boosted with χ9241 (pYA4088) and χ12358 (pYA4088) were challenged i.p. with 2.0 × 10^4^ CFU (50 times of LD_50_) of wild type *S. pneumoniae* WU2. No mice in the negative control groups immunized with χ9241 (pYA3493) survived. However, both of the χ9241 (pYA4088) and χ12358 (pYA4088) strains provided significant protection against the challenge. Moreover, there was no difference in the level of protection afforded by the RASVs and *wec* operon mutants, suggesting that deletion of the *wec* operon had no influence on the protective efficacy (Table 2).

**Table 2:** Oral immunization with the PspA-expressing *S.* Typhimurium χ9241 (pYA4088) vaccine and χ12358 (pYA4088) protected BALB/c mice against a challenge with the virulent *S. pneumoniae* strain WU2

Vaccine	PspA expression^a^	Total mice/group	Survival/total^b^	Percent protection^b^
χ12358 (pYA4088)	+	12	4/12	33%
χ9241 (pYA4088)	+	12	4/12	33%
χ9241 (pYA3493)	–	12	0/12	0%

^a^ +, PspA expressed; −, PspA not expressed

^b^ Mice were challenged with 4 × 10^4^ CFU of *S. pneumoniae* WU2 (50 times LD_50_) 4 weeks after the second immunization

## Discussion

Most mutations related to the loss of ECA are restricted to a single mutation, which are limited by the high possibility of reversible virulence and redundant genes presence in the genome, leading to a waste of energy and nutrients necessary for bacterial growth [18, 21]. In this work, we constructed ECA-deficient *S.* Typhimurium mutants by deleting the entire ECA operon from *wecA* to *wecG* and evaluated the mutant strain for its virulence and ability to be used for heterologous antigen delivery. Our studies proved the susceptibility of mutants carrying the ECA operon deletion to the bile salt deoxycholate, which is consistent with a previous report that mutations of *wecA* genes in *Salmonella* enhance the sensitivity to bile salts [11] . We also observed significant attenuation of the ECA operon mutants and persistent loading in mouse organs, consistent with previous reports [18, 21].

When assessing the LD_50_ and bacterial loading of the ECA-deficient strain in mice, we observed meningitis-like symptoms (head tilt, spinning and unidirectional motion) in a small portion of the mice that were orally administered a dose higher than 10^8^ CFU of the ECA-deficient strain χ12357. While the ECA-deficient strain can still potentially be used to develop vaccines [21], to ensure the safety of live *Salmonella* vaccines, additional genetically unlinked mutations should be included in the vaccine strain to further attenuate these potential side effects. Therefore, Δ (*wecA*-*wecG*)-*6* was combined with the Δ*pabAB* mutation in strain χ9241 to create vaccine strain χ12358 to evaluate the effects of ECA deficiency on immunogenicity by introducing plasmid pYA4088, which directs the synthesis of the heterologous pneumococcal protective antigen PspA [23, 37]. The obtained vaccine strain χ12358 (pYA4088) constituted a regulated delayed antigen synthesis system. In the presence of arabinose, the LacI protein prevented PspA expression by binding to the P_trc_ promoter on pYA4088; while this strain invades and colonizes host tissues by oral immunization, LacI ceases to be synthesized due to the absence of arabinose and PspA is expressed [38].

Mice immunized with the ECA-deficient vaccine χ12358 (pYA4088) elicited an anti-PspA IgG response, but the levels were significantly lower than that in mice immunized with χ9241 (pYA4088) (Fig. 4). The low elicited immune response by χ12358 (pYA4088) may ascribe to its greater attenuation and low colonization in mouse tissues compared with χ9241 (pYA4088) because these phenomena were also observed in the vaccine strain lacking the full-length O-antigen [23, 37]. Therefore, it is critical for live vaccine development to achieve a proper balance between attenuation and immunogenicity through the use of new vaccine development technologies such as regulated delayed in vivo attenuation and regulated delayed in vivo antigen synthesis, which enables vaccines to efficiently colonize host tissues, thereby inducing a sufficient immune response against protective antigens synthesized in the vaccine vectors [29, 39]. The key strategy for achieving regulated delayed in vivo attenuation relies on regulating essential gene expression by replacing the promoter of a gene of interest with the regulated promoter, such as arabinose-regulated *araC* P_BAD_ [30, 39]. Our previous results already demonstrated that this strategy is beneficial for inducing higher immune responses against vector delivered antigens [23, 37]. Meanwhile, a *Salmonella* mutant (Δ*rfbB6* Δ*rffG7* Δ*pagL73*::TT *araC* P_BAD_*rfbB*-3), achieving the simultaneous tight regulation of both LPS and ECA synthesis in vitro by exogenously supplied arabinose, was obtained in our lab [26], and its capability to deliver heterologous antigens and induce immunogenicity in the χ 9241 background vaccine strain will be evaluated in the future.

In mice, the direction of IgG (IgG1 vs IgG2a) switching can reflect the polarization of the Th cell response [40]. We compared the levels of two isotype subclasses of anti-PspA IgG1 and IgG2a in this study. Surprisingly, we found that the levels of the two subclasses of IgG1 and IgG2a isotype antibodies stimulated by the ECA-deficient mutant strain and its attenuated *Salmonella* patent strain showed different trends. The level of anti-PspA IgG1 isotype antibodies in mice immunized with the ECA-deficient vaccine were significantly higher than those with χ9241 (*P* < 0.001) (Fig. 4), and the IgG2a levels were significantly lower than those with χ9241 (pYA4088) (P < 0.001) (Fig. 4). While it is believed that murine IgG1 is strongly dependent on the IL-4 and Th2 type response, and it has a modest capacity to bind C1q of the complement system due to its weak binds to FcγRIIB and FcγRIII, and that Th17-mediated immunity is essential for protection against pneumococcal colonization and for the clearance of colonization [41, 42], the enhanced production of IgG1 should be beneficial for preventing against pneumococcal infection and clearing extracellular pathogens because the equivalent level of protection was obtained with both the ECA-deficient strain χ12358 (pYA4088) and χ9241 (pYA4088), in spite of the significantly lower IgG levels induced by the ECA mutant. In addition, in this study, we employed live *Salmonella* as the carrier to induce an immune responses against pneumococcal surface protein antigens in a pneumococcal animal model; thus, the conclusions obtained from purified pneumococcal protective proteins or polysaccharides may not be suitable for our study as live recombinant *Salmonella* vaccines could stimulate all arms of the immune response, including mucosal, humoral, and cellular immunities [43, 44].

## Conclusions

In summary, we examined the relationship between *S.* Typhimurium ECA and biological characteristics, virulence, colonization, immunogenicity using an ECA-negative mutant in conjunction with other attenuated mutants as vehicles for heterologous antigen delivery. Our work demonstrates that ECA affects virulence and benefits the Th2 immunity of *Salmonella* Typhimurium. Additionally, the ECA-deficient mutant strain of *S.* Typhimurium was seriously attenuated after oral infection [18]. In addition, our data presented here also show that the ECA mutant, in the context of an attenuated vaccine strain, induces a weak, slightly switching IgG1 immune response but still confers the same protection to *S. pneumoniae* WU2. Therefore, based on the above results, we speculate it is feasible to use a reversibly ECA mutant mode to design future *Salmonella* vaccine strains for heterologous protective antigens.

## Methods

### Bacterial strains, plasmids, media and experimental animals

The bacterial strains and plasmids used in this study are listed in Table 3. Bacterial strains were maintained in LB broth plus 20% glycerol at − 80 °C. *S.* Typhimurium cultures were grown at 37 °C in LB broth or on LB agar. Diaminopimelic acid (DAP) (50 μg/ml) was added to grow the Δ*asd* strains [45]. LB agar containing 5% sucrose was used for *sacB* gene-based counter-selection in the allelic exchange experiments [46]. Chloramphenicol (Cm, 25 μg/ml) was added to the media when needed. The seven-week-old female BALB/c mice used in this experiment were purchased from Dashuo Experimental Animal Ltd. (Chengdu, China). Mice were acclimated for 7 days after arrival before starting the experiments. All animal procedures were approved by the Southwest University Animal Care and Use Committee.

**Table 3:** Bacterial strains and plasmids

Strain or plasmid	Description of the genotype or relevant characteristics	Source or reference
*S.* Typhimurium stains		
χ3761	Wild type UK-1	[31]
χ9241	Δ*pabA1516* Δ*pabB232* Δ*asd*A*16* Δ*araBAD23* Δ*relA198*::*araC* P_BAD_*lacI* TT	[32]
χ12357	Δ (*wecA*-*wecG*)-6, Delete whole ECA operon in χ3761	This study
χ12358	Δ (*wecA*-*wecG*)-6, Delete whole ECA operon in χ9241	This study
*S. pneumoniae* strains	WU2, Wild-type virulent, encapsulated type 3	[34]
Plasmids		
pYA4278	*SacB* mobRP4 R6K ori Cm^+^, pRE112-T-vector	[24]
pYA5493	Delete whole ECA operon	This study
pYA3493	Plasmid Asd^+^; pBR*ori* β-lactamase signal sequence-based periplasmic secretion plasmid	[35]
pYA4088	852-bp DNA encoding the α-helical region of PspA from aa 3–285 in pYA3493	[27]

### Plasmids and mutant strain construction

The primers used in this study are listed in Table 4. DNA manipulations were carried out as described [47]. Transformation of *E. coli* and *S.* Typhimurium was achieved by electroporation. Transformants were selected on LB agar plates containing the appropriate antibiotics. For the ECA operon deletion experiments, Doperon-1F/Doperon-1R and Doperon-2F/Doperon-2R primers were used to amplify approximately 400-bp fragments upstream and downstream of the ECA operon from the χ3761 genome, respectively. The two fragments were then joined by PCR using Doperon-1F and Doperon-2R primers. Terminal A was simultaneously added to both ends of the resulting PCR product by LA-Taq enzyme (Takara, Japan) and ligated to pYA4278 to generate plasmid pYA5493. The mutation was introduced into *S.* Typhimurium χ3761 by allelic exchange using suicide vectors pYA5493 to generate χ12357. The mutation was also introduced into *S.* Typhimurium vaccine strain χ9241 to yield strain χ12358.

**Table 4:** Primers used in this work

Primer	Sequence from 5’to 3´
Doperon-1F Doperon-1R Doperon-2F Doperon-2R	CCATCCGATGGGTGAAATTG AGGGAGACCTGCAGGACGCCAGCTCCTAGAAGAGAC TGGCGTCCTGCAGGTCTCCCTTCTGATGACTTGAG GATATACGCCAGCAGCACTG

### ECA western blot and LPS analyses

ECA was prepared as previously described [48]. ECA phenotypes were characterized by immunoblotting. Bacteria grown overnight on LB plates were pelleted and washed twice with physiological saline, re-suspended in 100 μl of lysis buffer (0.065 M pH 6.8 Tris-HCl, 5% β-mercaptoethanol, 2% SDS, 10% glycerol, 0.05% bromophenol blue) and heated at 100 °C for 10 min, and then incubated overnight at 60 °C with proteinase K to a final concentration of 1.6 μg/ml. Samples were boiled again for 10 min. ECA samples were separated on 12% Tricine-SDS-PAGE gels, and transferred to nitrocellulose membranes. Membranes were probed with a 1:1000 dilution of murine anti-ECA monoclonal antibody mAb898 [48]. Blots were developed using a BCIP/NBT chromogenic substrate kit and imaged using a Gel Doc TM XR+ with Image Lab™ Software (BIO-RAD, USA). The LPS and silver staining analyses were based on previously described protocols [49]. Briefly, cells from overnight cultures were lysed and boiled for 10 min, and then incubated with proteinase K for 1 h at 37 °C. Subsequently, 10 μl of each sample was separated by SDS-PAGE (12%), and the gels were stained with silver nitrate. For the immunoblotting experiments, the isolated ECA samples were separated by sodium dodecyl sulfate-polyacrylamide gel electrophoresis (SDS-PAGE), the resulting bands were transferred to nitrocellulose membranes, and the nitrocellulose membranes were probed with the murine anti-ECA monoclonal antibody mAb898 at a 1:1000 dilution [48].

### Motility assays

LB plates containing 0.3% agar were inoculated with each strain and dried at room temperature for 2 h. For the motility assays, three microliters of fresh bacterial liquid cultures were spotted onto the middle of plates and incubated at 37 °C for 6 h. The diameter of the colonies was measured. The experiments were repeated three times.

### DOC sensitivity assay

Bacteria were grown until they reached an OD_600_ of 0.8–0.9. Dilutions from exponential cultures of each strain were spread on LB plates supplemented with 1% DOC [11]. Three-microliter portions of the appropriate dilutions of the exponential cultures of the wild-type and mutant strains were incubated overnight at 37 °C in LB plates with or without 1% DOC. To precisely calculate the survival rate in DOC, 100 μl of a 100-fold dilution of the bacterial cultures with an OD_600_ of 0.8 were transferred to a new tube, DOC was added to final concentration of 10 mg/ml, and then incubated for 1 h at 37 °C. The samples were then diluted to the appropriate concentration, and then 100 μl of the dilutions were spread onto LB plates, followed by overnight incubation at 37 °C. The assays were repeated three times.

### Determination of the LD_50_ and colonization in mice

Following our standard procedures, the oral 50% lethal dose (LD50) was determined [37]. Bacteria were grown statically overnight at 37 °C in LB broth, diluted to 1:50 in fresh media, and grown with aeration at 37 °C. When the cultures reached an OD600 of 0.8–0.9, they were harvested at room temperature by centrifugation at 4000 rpm, washed once, and normalized to the required inoculum density in buffered saline gelatin (BSG) by adjusting the suspension to the appropriate OD600 value. Groups of five mice each were infected orally with 20 μl containing various doses of S. Typhimurium χ3761 or the mutant χ12357, ranging from 1.0 × 103 CFU to 1.0 × 109 CFU. Mice were observed for 4 weeks post-infection, and deaths were recorded daily. To evaluate colonization, mice were inoculated orally with 20 μl of BSG containing 1 × 109 CFU of the wild type and mutant strains. Peyer’s patches (PP), spleen and liver tissues were harvested at days 3, 6, 9, 12, 15 and 20 post-infection. The standard procedure for determining the bacteria colonization in PP, spleen and liver in the mouse was described in our previous publications [23, 37]. Briefly, we collected all the PP from the small intestinal surface, spleen from each mouse, or cut a slice of mouse liver into 1.5 ml tube. 1 ml BSG was added to the tube containing PP, and spleen or a slice of liver were measured the weight and extra BSG were added to a total volume of 1 ml. The samples were homogenized and plated onto LB plates to determine the number of viable bacteria after appropriate dilutions. The means of pooled PP were calculated in each mouse as bacterial colonization number in PP, and the bacterial number of liver and spleen were calculated as bacterial colonization number in spleen and liver.

### Western blot analyses

To test the expression level of the heterologous antigen (PspA) delivered by the *S*. Typhimurium vaccine carrier χ9241 and its ECA operon mutant strain, western blotting analyses were performed. Two proteins, LacI and GroEL, were used as the controls. The standard western blotting procedure were followed to visualize protein expression in bacteria [37], Briefly, the proteins were separated by SDS-PAGE and transferred to nitrocellulose membranes. The membranes were blocked with 3% skim milk in 10 mM Tris and 0.9% NaCl (pH 7.4) and incubated with rabbit polyclonal antibodies specific for PspA, LacI or GroEL (Sigma, St. Louis, USA) [35]. Later, the membranes were washed three time using 10 mM Tris (pH 7.4) with 0.05% Tween 20, and inoculated in the AP-conjugated goat anti-rabbit immunoglobulin G (IgG) (Sigma) for 1 h at room temperature. Last, the membranes were rinsed three time with 10 mM Tris (pH 7.4) with 0.05% Tween 20 and were visualized by addition of reactive substrate of BCIP-NBT solution (Sigma), the reaction was stopped by washing the blots with large volumes of pure water when the bands developed clearly.

### Immunogenicity of the vaccine strains in mice

The attenuated vaccine strains χ9241 (pYA4088), χ12358 (pYA4088) and χ9241 (pYA 3493) were cultured in LB with and without 0.1% arabinose and diluted with BSG for immunization following previous described procedures [37]. Briefly, BALB/c mice were orally inoculated with 20 μl of BSG containing approximately 1.0 × 10^9^ colony forming units (CFU) of each strain suspension prepared as described above, and then boosted on day 28 with the same dose of the same strain. The vaccinated mice were monitored for symptoms and mobility during the immunization period. Blood were obtained by mandibular vein puncture at biweekly intervals. Serum samples were obtained and stored at − 80 °C for ELISA analysis.

### ELISA analysis of serum antibodies

To measure the levels of antibodies triggered by the vaccine strains in the serum, ELISA assays were performed with serum antibodies against *E. coli* recombinant PspA (rPspA) and outer membrane proteins (SOMP) as previously described [37]. Briefly, rPspA and SOMP proteins of *S*. Typhimurium were prepared and purified as described previously [35]. Polystyrene 96-well flat-bottom microtiter plates were coated with 100 ng/well of purified rPspA or SOMPs in the coating buffer (50 mM Na_2_CO_3_, 50 mM NaHCO_3_, 0.1% sodium azide, pH 9.6), and 100 ng/well of goat anti-mouse IgG (H + L) in PBS was applied with 100 μl volumes to coat the two lines in 96-well plates for standard curve drawing. The coated plates were incubated overnight at 4 °C, followed by blocking with PBS containing 10% FBS (Sigma, USA) for 1 h at room temperature. A 100 μl volume of serum diluted sample or mouse IgG (BD Pharmingen) for the standard curve were added to individual wells in triplicate and incubated for 1 h at 37 °C. Plates were treated with biotinylated goat anti-mouse IgG, IgG1 or IgG2a (Southern Biotechnology). Wells were developed with a streptavidin-alkaline phosphatase conjugate (Southern Biotechnology), followed by *p*-nitrophenylphosphate substrate (Sigma) in diethanolamine buffer (pH 9.8). Color development (absorbance) was recorded at 405 nm using a microplate reader. The standard curve was drawn using Curve Expert, and the serum antibody concentration was calculated using the standard curve.

### Pneumococcal challenge

The protective efficacy of immunization with the attenuated *Salmonella* strains expressing PspA was assessed. Vaccinated mice at day 56 were challenged with 2 × 10^4^ CFU of *S. pneumoniae* WU2 in 200 μl of BSG by intraperitoneal (i.p.) injection and monitored and recorded daily for 15 days [34].

### Statistical analysis

The antibody titer data are expressed as the geometric means, and the relative immunoreactivity is expressed as an arithmetic mean. The means were evaluated by two-way analysis of variance and chi-square test for multiple comparisons among the groups. A *P* value of <0.05 was considered statistically significant.

## Supplementary information

**Additional file 1: Figure S1.** The expression of PspA and lacI. The western blots showed the express of PspA in strains χ12358 (pYA4088), χ9241 (pYA4088) and χ9241 (pYA3493). The strains were cultured in LB broth with (+) or without (−) 0.1% arabinose overnight at 37 °C. Equal numbers of cells from each strain were pelleted, suspended in protein loading buffer and boiled. Equal volumes were separated on SDS-PAGE in triplicate gels. Each gel was transferred to nitrocellulose and reacted with polyclonal antibody specific for PspA, LacI and GroEL, respectively. GroEL was used as a standardization marker.

## Data Availability

Not applicable.

## Abbreviations

ECA: Enterobacterial common antigen
LPS: Lipopolysaccharide
DOC: Deoxycholate
DAP: Diaminopimelic acid
BSG: Buffered saline gelatin
SDS-PAGE: Sodium dodecyl sulfate-polyacrylamide gel electrophoresis
UPEC: Uropathogenic *E. coli*
RASV: Recombinant attenuated *Salmonella* vaccine
RDES: Regulated delayed expressed system
OMP: Outer membrane proteins
BCIP-NBT: 5-bromo-4-chloro-3-indolylphosphate-nitroblue tetrazolium solution
