# Supplementary figures and images for: Effect of deletion of gene cluster involved in synthesis of Enterobacterial common antigen on virulence and immunogenicity of live attenuated Salmonella vaccine when delivering heterologous Streptococcus pneumoniae antigen PspA
Source: BMC Microbiol. 2020 Jun 8;20:150. doi: 10.1186/s12866-020-01837-0 (PMC7278252; doi:10.1186/s12866-020-01837-0)

## Slide 1
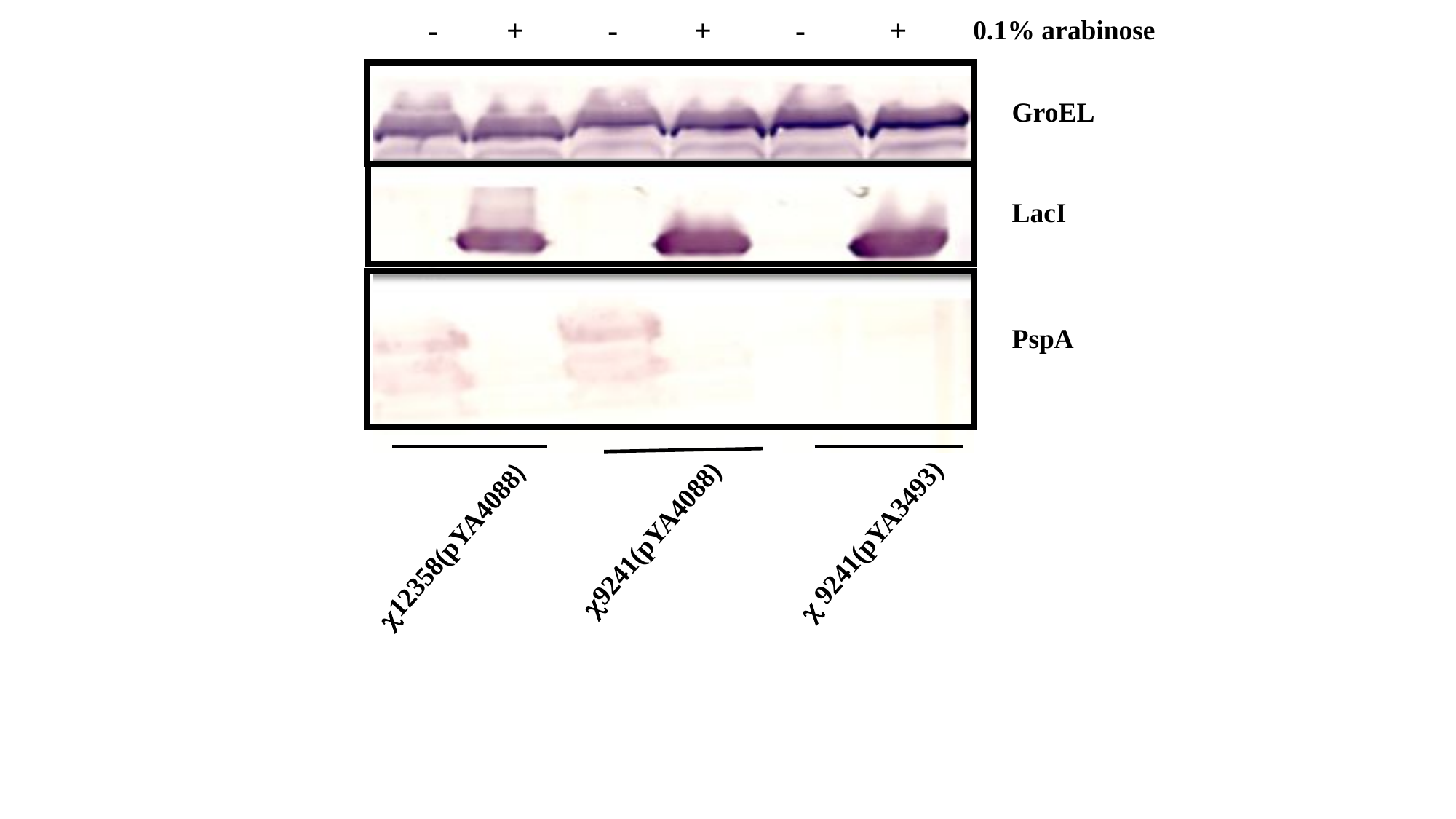

- + - + - +
0.1% arabinose
GroEL
LacI
PspA
9241(pYA4088)
 9241(pYA3493)
 12358(pYA4088)

Supplement: Supplementary file 1 — Additional file 1: Figure S1. The expression of PspA and lacI. The western blots showed the express of PspA in strains χ12358 (pYA4088), χ9241 (pYA4088) and χ9241 (pYA3493). The strains were cultured in LB broth with (+) or without (−) 0.1% arabinose overnight at 37 °C. Equal numbers of cells from each strain were pelleted, suspended in protein loading buffer and boiled. Equal volumes were separated on SDS-PAGE in triplicate gels. Each gel was transferred to nitrocellulose and reacted with polyclonal antibody specific for PspA, LacI and GroEL, respectively. GroEL was used as a standardization marker. [file 12866_2020_1837_MOESM1_ESM.pptx]
